# Para-hydrodynamics from weak surface scattering in ultraclean thin flakes

**DOI:** 10.1038/s41467-023-37966-z

**Published:** 2023-04-22

**Authors:** Yotam Wolf, Amit Aharon-Steinberg, Binghai Yan, Tobias Holder

**Affiliations:** Department of Condensed Matter Physics, Weizmann Institute of Science, Rehovot, Israel

**Keywords:** Electronic properties and materials, Surfaces, interfaces and thin films

## Abstract

Electron hydrodynamics typically emerges in electron fluids with a high electron–electron collision rate. However, new experiments with thin flakes of WTe_2_ have revealed that other momentum-conserving scattering processes can replace the role of the electron–electron interaction, thereby leading to a novel, so-called para-hydrodynamic regime. Here, we develop the kinetic theory for para-hydrodynamic transport. To this end, we consider a ballistic electron gas in a thin three-dimensional sheet where the momentum-relaxing (lmr) and momentum-conserving (lmc) mean free paths are decreased due to boundary scattering from a rough surface. The resulting effective mean free path of the in-plane components of the electronic flow is then expressed in terms of microscopic parameters of the sheet boundaries, predicting that a para-hydrodynamic regime with lmr ≫ lmc emerges generically in ultraclean three-dimensional materials. Using our approach, we recover the transport properties of WTe_2_ in the para-hydrodynamic regime in good agreement with existing experiments.

## Abstract (web summary)

Classical hydrodynamics applies to electron fluids as well, provided the system has a high electron–electron collision rate. Now, model calculations show that regimes in which other scattering processes are at play can explain hydrodynamic electron transport in thin flakes of the 2D material WTe_2_.

## Introduction

The viscous flow of an interacting electron fluid was predicted a long time ago^1^; however, experimental evidence for it has remained scarce for many decades^2^. The advent of ultraclean quantum materials with low carrier density^3–5^ yielded a growing number of cases demonstrating viscous electron flow in the last few years^6–11^. Moreover, it has even become possible to establish ballistic and viscous flow profiles using spatially resolved techniques^12–16^. Among the coveted properties of hydrodynamic flow is, for example, a negative nonlocal resistance^17–20^ as well as other nonlocal transport signatures^21–27^. However, the observation of vortical flow (electron whirlpools) still remained elusive^28–31^. This situation was upended very recently when a high-fidelity, spatially resolved experiment^32^ in ultraclean WTe_2_^33,34^ could unambiguously demonstrate hydrodynamic vortical flow, clearly excluding a ballistic origin of the observed whirlpool pattern.

The observation of this hydrodynamical vortical flow is remarkable for two additional reasons. First, the device is not an effective two-dimensional system but a thin (thickness *d* = 48 nm, width *w* = 550 nm), three-dimensional flake exfoliated and fabricated from WTe_2_ flakes with a very large bulk mean free path *ℓ* ≈ 20 μm. Second, at the measured temperature *T* = 4.5 K, the electron–electron interaction leads to a momentum-conserving mean free path *ℓ*_*ee*_ ≈ 10 μm ≫ *w*. This means that the sample cannot be in the hydrodynamic regime, which is characterized by the condition that *ℓ*_*ee*_ ≪ *w* ≪ *ℓ*^1^.

**Fig. 1:**
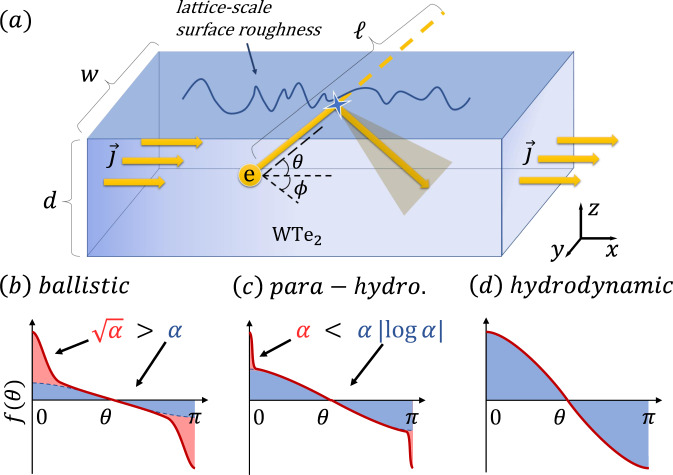
Phenomenology of para-hydrodynamics. **a** The current flows through a thin slab of thickness *d* ≪ *w* ≪ *ℓ*. Electrons scatter at the microscopically rough top and bottom surfaces with incident angle *θ*. Most trajectories are reflected almost specularly, leading to angular diffusion in *θ*, while few trajectories scatter randomly, thus dissipating momentum. **b**–**d** Comparison of the non-equilibrium distribution functions *f*(*θ*) in different flow regimes (red curve). An approximate cosine that inscribes *f*(*θ*) for steep angles *θ* ≠ 0, *π* is shown as a blue shaded area, whereas the red shaded area indicates grazing trajectories (*θ* ≈ 0, *π*). Different parts of *f*(*θ*) have dissimilar scale dependencies in terms of the ratio *α* = *d*/2*ℓ* ≪ 1. For ballistic flow, *f*(*θ*) decays strongly upon approaching *θ* = *π*/2, while for hydrodynamic flow, it assumes a smooth cosine form everywhere. The intermediate para-hydrodynamic regime carries signatures of both ballistic and hydrodynamic flow, but the smooth part of *f*(*θ*) is logarithmically larger than the ballistic part, thus leading to an effectively hydrodynamic current in the limit *α* → 0.

Based on the unusual properties of WTe_2_, the authors in ref. ^32^ suggested that an effectively hydrodynamic flow could instead be induced by almost specular (and thus predominantly momentum-conserving) scattering from the top and bottom surfaces of the three-dimensional WTe_2_ sheet, a mechanism termed para-hydrodynamics (cf. Fig. 1a). However, no kinetic theory has been put forward which would explain how such a novel type of hydrodynamic flow emerges microscopically.

In this letter, we consider a kinetic theory for the in-plane flow of an electron fluid in a thin, three-dimensional slab that takes into account weak boundary scattering from the rough top and bottom surfaces. We demonstrate that this setting naturally leads to para-hydrodynamic flow in thin three-dimensional devices as long as the bulk mean free path *ℓ* of the material is very large compared to the device thickness *d*. The proposed scattering model is generic, with the only parameters being the amplitude and correlation length of the surface roughness.

The mechanism is depicted schematically in Fig. 1b–d. The emergence of the para-hydrodynamic regime is a result of the conversion of rarely colliding trajectories that impact the top and bottom surfaces at a grazing angle (i.e., ballistic flow) into trajectories that scatter often and at a steep angle from the surface (hydrodynamic flow). This conversion happens due to the slow angular diffusion of the scattered trajectories. The redistribution of statistical weight means that the distribution function and, thus, also the current predominantly resembles hydrodynamic transport. Therefore, while it is not possible to describe the three-dimensional distribution function using a Stokes–Ohm hydrodynamic approach, the in-plane components of the flow velocity exhibit relaxation properties which resemble viscous flow. Specifically, we find that the effective in-plane momentum-relaxing (*ℓ*_*mr*_) and momentum-conserving (*ℓ*_*mc*_) mean free paths are related as ${\ell }_{mr}/{\ell }_{mc}\propto \log (\ell /d)\, > \,1$. Since the conversion of forward trajectories into steep trajectories is bound to happen whenever angular diffusion is present in the boundary scattering, the only reason why the phenomenon of para-hydrodynamics has so far remained elusive seems to be the logarithmically slow enhancement of this effect with increasing mean free path. In particular, our model posits that neither the precise surface roughness nor the material itself sensitively affects whether a para-hydrodynamic transport regime can emerge in a given material. Instead, the para-hydrodynamic regime merely requires a very large fineness ratio *ℓ*/*d*, combined with an appropriate choice of the width *w* of the slab so that *ℓ*_*mr*_ ≫ *w* ≫ *ℓ*_*mc*_. These conditions were fulfilled in the experiment of ref. ^32^, which was done with high-quality samples where *ℓ*/*d* > 500.

## Results

### Absence of e–e interactions at low T

**Fig. 2:**
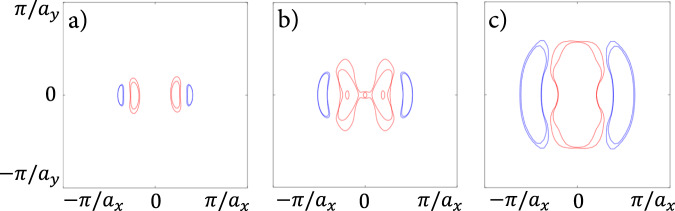
Candidate Fermi surfaces for WTe_2_, with electron (hole) pockets in blue (red). **a** Highly anisotropic pockets, **b** large pockets, and **c** very small pockets, corresponding to a semimetallic state. We find that the effective electron–electron mean free path does not depend sensitively on the choice of Fermi surface.

It has been argued^16,32^ that WTe_2_ cannot be in the hydrodynamic regime below 20 K. However, estimates for the effective electron–electron mean free path *ℓ*_*ee*_ may vary considerably depending on the employed band structure model and other details of the calculational approach. Therefore, before invoking a surface mechanism, we briefly comment that bulk scattering is unequivocally too weak to matter in these mesoscopic devices. To this end, consider the electronic self-energy for three qualitatively different but realistic candidate band structures of the three-dimensional phase of WTe_2_. The proposed Fermi surfaces describe (i) a Weyl semimetal phase with very small pockets, (iii) a phase with highly anisotropic pockets, and (iii) a relaxed phase with large pockets (Fig. 2). Employing standard methods to calculate the imaginary part of the self-energy due to the screened Coulomb interaction^35^ and using a fine momentum grid (cf. Supplementary Information), we obtain the estimates ${\ell }_{ee}^{(i)}=19{{{{{{{\rm{\mu m}}}}}}}}$, ${\ell }_{ee}^{(ii)}=22{{{{{{{\rm{\mu m}}}}}}}}$ and ${\ell }_{ee}^{(iii)}=155{{{{{{{\rm{\mu m}}}}}}}}$ at *T* = 4.5 K. Therefore, we conclude that details of the calculation, and differing starting assumptions about the qualitative shape of the Fermi pockets do not substantially affect the electron–electron scattering rate, soundly excluding bulk mechanisms as the source of the hydrodynamic flow. Note that ref. ^16^ additionally considered phonon-assisted electron–electron interactions, but they are likewise too weak at low T.

### Boundary scattering model

Due to the nature of the para-hydrodynamic transport regime, the derivation cannot rely on a hydrodynamic treatment but has to start from a Boltzmann transport approach. In the following, we consider the nearly ballistic flow in a three-dimensional rectangular geometry where *d* ≪ *w* ≪ *ℓ* (cf. Fig. 1). In the kinetic approach, the distribution function is denoted by *f*(**r**, **k**) at real-space position **r** and for momentum **k** on a spherical Fermi surface. While the material in consideration has a complicated, non-spherical Fermi surface, the relevant Fermi surface quantity in the Boltzmann equation is the Fermi velocity, which is indeed relatively isotropic. The Boltzmann equation in the steady state is

1 $${{{{{{{{\bf{v}}}}}}}}}_{F}\cdot {\nabla }_{{{{{{{{\bf{r}}}}}}}}}\,f+e{{{{{{{\bf{E}}}}}}}}\cdot {\nabla }_{{{{{{{{\bf{k}}}}}}}}}\,f={{{{{{{{\mathcal{I}}}}}}}}}_{0}(\,\,f)+{{{{{{{{\mathcal{I}}}}}}}}}_{b}(\,\,f).$$

Here, **v**_*F*_ is the Fermi velocity, *e* is the electron charge, **E** = (*E*_*x*_, 0, 0) is the electric field and ${{{{{{{{\mathcal{I}}}}}}}}}_{0}(f)=|{{{{{{{\bf{v}}}}}}}}|(f-{f}_{0})/\ell$ is the bulk collision integral in relaxation time approximation. ${{{{{{{{\mathcal{I}}}}}}}}}_{b}(f)$ is the collision integral due to boundary scattering from the top and bottom surfaces. Because the thickness *d* of the flake is much smaller than its width *w*, we can neglect the spatial dependence along the width of the channel. We note that this approximation becomes exact when the in-plane boundaries at *y* = ±*w*/2 are completely specular, in which case the distribution function is *y*-independent. We parametrize $f({{{{{{{\bf{r}}}}}}}},{{{{{{{\bf{k}}}}}}}})-{f}_{0}=Ah(z,\theta )\cos \theta \cos \phi$, choosing −*d*/2 ≤ *z* ≤ *d*/2 along the third dimension, −*π*/2 ≤ *ϕ* ≤ *π*/2 as the angle in the plane, and −*π* < *θ* ≤ *π* as the out-of-plane angle, with *θ* = *ϕ* = 0 pointing along +*x* (cf. Fig. 1). The dimensionless coefficient *A* = −(∂_*ϵ*_ *f*_0_)*eE*_*x*_*ℓ* is chosen such that the solution *h* becomes normalized (*h*(*z*, *θ*) = 1) when boundary scattering is absent, associated with the bulk current density ${j}_{0}=\frac{e{v}_{F}}{32\pi }\int{d}^{3}{{{{{{{\bf{k}}}}}}}}A$.

We now construct *h*(*z*, *θ*), which solves Eq. (1) for boundary scattering from a rough surface. The microscopic process of boundary scattering has been studied for many decades^36–43^. It is common to rewrite the boundary collision integral in terms of boundary conditions using a specularity parameter *R*_*θ*_ which may depend on the angle of incidence *θ*. Here, *R*_*θ*_ = 0 corresponds to completely diffuse scattering, while *R*_*θ*_ = 1 is fully specular. Using this parameter, one can express the reflected part of the distribution function *h*(*d*/2, −∣*θ*∣) at the top surface in terms of the incident one as *h*(*d*/2, −∣*θ*∣) = *R*_*θ*_*h*(*d*/2, ∣*θ*∣), and vice versa at the bottom surface it is *h*(−*d*/2, ∣*θ*∣) = *R*_*θ*_*h*(−*d*/2, −∣*θ*∣). However, using a true 2D scattering cross section to describe a boundary scattering event, one has to go back to the full distribution function, expressing the reflected *f* ^>^ in terms of the incident *f* ^<^ by an integral condition^38^

2 $${f}^{\ > \ }({{{{{{{\bf{k}}}}}}}})={f}^{ < }({{{{{{{\bf{k}}}}}}}})+{k}_{z}\,\,{\int}_{F{S}^{{\prime} }}\,{d}^{2}{k}^{{\prime}} {k}_{z}^{{\prime}}W({{{{{{{\bf{k}}}}}}}}-{{{{{{{{\bf{k}}}}}}}}}^{{\prime}})[\,\,{f}^{ < }({{{{{{{{\bf{k}}}}}}}}}^{{\prime}})-{f}^{ < }({{{{{{{\bf{k}}}}}}}})]$$

where *W*(**k**) is the correlation function of the surface scattering potential and the integral runs over the half-sphere (FS’) of the Fermi surface, which corresponds to trajectories incident to the boundary. We henceforth employ a generic Gaussian-correlated scattering potential^38^, defined as $W({{{{{{{\bf{k}}}}}}}})=\pi {a}^{2}{b}^{2}{e}^{-{{{{{{{{\bf{k}}}}}}}}}^{2}{b}^{2}/4}$, with potential depth *a* and correlation length *b*.

Historically, Eq. (2) has been solved in two limiting cases, for grazing angles *θ* ≈ 0 and for steep angles of incidence, *θ* ≈ *π*/2^38^. For grazing angles, the distribution function changes more rapidly than the scattering cross section *W* and one obtains a standard form of the boundary in terms of the angle-dependent specularity (1 − *q*∣*θ*∣) with parameter $q=4\sqrt{\pi }{{\Gamma }}(\frac{3}{4}){a}^{2}{k}_{F}^{3/2}/\sqrt{b}$. Conversely, for steep angles, the scattering potential *W* changes faster than the distribution function. Using a saddle-point approximation therefore yields a Fokker–Planck equation in the angle of the form $h(d/2,-|\theta|)={\hat{O}}_{\theta }h(d/2,|\theta|)$ with the operator of the angular diffusion on the Fermi surface being ${\hat{O}}_{\theta }=Q{\sin }^{2}\theta ((2\cot \theta -\tan \theta ){\partial }_{\theta }+{\partial }_{\theta }^{2})$, where *Q* = 8*a*^2^/*b*^2^. To our knowledge, no attempts have been made to treat the scattering for both limits, *θ* ≈ 0 and *θ* ≈ *π*/2, in a unified framework. However, to capture the para-hydrodynamic behavior, we seek a solvable description that holds for all angles of incidence. Indeed, as we demonstrate next, the description for general angle *θ* is absolutely vital to capture the parametric dependencies of the para-hydrodynamic flow correctly.

In the spirit of Matthiessen’s rule, we propose to add both scattering limits as two different types of scattering processes, which yields for the distribution function at the upper boundary of the slab the boundary condition

3 $$h(d/2,-| \theta |)=({R}_{\theta }+{\hat{O}}_{\theta })h(d/2,| \theta |).$$

Here, we introduced the specularity parameter ${R}_{\theta }=(1-q\sin|\theta|)$, which is the periodic extension of the previously mentioned specularity at small angles. Note that both scattering types can be combined safely because for *θ* = 0, *π*, the momentum-relaxing scattering vanishes (*R*_0_ = 1), meaning that the distribution function has no discontinuities anywhere. Equation (3) presents the key innovation for a unified treatment of boundary scattering.

The ordinary differential Eq. (3) exhibits several favorable properties. First, solutions for *q* = 0 and for any *Q* > 0 are non-dissipative, with distribution function *h*(*z*, *θ*) ≡ 1, corresponding to a bulk current profile without any stresses. This can be understood as follows. *Q* parameterizes the relative importance of angular diffusion due to scattering from the boundaries. However, without any momentum loss (i.e., unless *q* > 0), the angular diffusion of momentum will redistribute momenta equally into higher and lower momentum states, thereby conserving momentum exactly. Second, the differential equation becomes stiff both at *θ* = 0 and *θ* = *π*/2, both of which constitute singular points. Therefore, the resulting distribution function at either point singularly depends on the initial conditions at the respective other point. Indeed, we find that for *Q* ≫ *q*, the solution retains a singular dependence on *q* for all angles, even though *R*_*θ*_ is dominant over ${\hat{O}}_{\theta }$ only for shallow angles smaller than *d*/*ℓ*.

### Intermediate regime of para-hydrodynamics

Using a symmetric parametrization in terms of the characteristics of the motion^44^, the solution of the Boltzmann equation can be written as

4 $$h(z, \theta )={e}^{-z\csc \theta /\ell }c(\theta )+(1-{e}^{-z\csc \theta /\ell }).$$

**Fig. 3:**
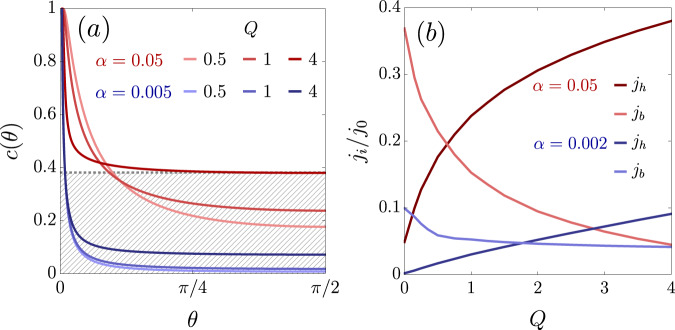
Normalized distribution function and currents in the two-fluid picture. **a** The distribution function *c*(*θ*) in the para-hydrodynamic regime, for *q* = 1, three different values of *Q* and two *α*. With increasing *Q*, more weight is accumulated at the steep trajectories away from *θ* = 0. For *α* = 0.05, *Q* = 4, the asymptotic value *c*(*π*/2) = *c*_1_ is indicated by a gray dashed line. In the two-fluid approximation, the current *j*_*h*_ stemming from the area below *c*_1_ (gray, hatched) is compared against the current *j*_*b*_ induced by the rest of the non-equilibrium distribution. **b** Comparison of the para-hydrodynamic (*j*_*h*_/*j*_0_) and ballistic (*j*_*b*_/*j*_0_) current density contributions in the two-fluid approximation for two values of *α*, where *j*_0_ denotes the bulk current density. For all *α* ≪ 1, the contribution of the para-hydrodynamic current increases with *Q*, quickly overtaking the ballistic contribution.

*c*(*θ*) is symmetric in *θ* and is determined by the modified boundary scattering condition. Inserting the general solution of Eq. (4) into (3), one obtains for 0 < *θ* < *π*/2

5 $${e}^{\alpha \csc \theta }c(\theta )+{h}_{in}^{-}=({R}_{\theta }+{\hat{O}}_{\theta })({e}^{-\alpha \csc \theta }c(\theta )+{h}_{in}^{+})$$

where *α* = *d*/2*ℓ* and ${h}_{in}^{\pm }=(1-{e}^{\mp \alpha \csc \theta })$. Solving Eq. (5) is rather involved (cf. Supplementary Information). Typical solutions for *q* = 1 and several values of *Q* are shown in Fig. 3a. For *Q* ≈ 0, the distribution function quickly decays with increasing angle, meaning that the current originates almost exclusively from long-lived trajectories in the forward direction (*θ* = 0). This is the expected behavior for ballistic flow. In contrast, for *Q* > 1, the profile changes qualitatively, with the trajectories at grazing angles being strongly suppressed, while the distribution function becomes a cosine (corresponding to $c(\theta )={{{{{{{\rm{const.}}}}}}}}$) for all other angles. This shape of the distribution function resembles a hydrodynamic distribution function. The flat part is characterized by the asymptotic value *c*_1_ = *c*(*π*/2), which by Taylor expansion in *θ* ≈ *π*/2, evaluates to

6 $${c}_{1}=1-\frac{q{e}^{\alpha }-2Q{c}^{\prime{\prime}}(\frac{\pi }{2})}{{e}^{2\alpha }-1+q+2Q\alpha }$$

7 $$\approx \frac{2Q{c}^{\prime{\prime}}(\frac{\pi }{2})+(2-q+2Q)\alpha }{q+2(1+Q)\alpha }\quad{{{{{{{\rm{for}}}}}}}}\quad\alpha \to 0,$$

where ${c}^{\prime{\prime}}(\pi /2)=\frac{1}{2}{\partial }_{\theta }^{2}c(\theta ){|}_{\theta=\pi /2}$. In the ballistic regime (*Q* = 0), we find numerically that *c*^*″*^(*π*/2) ∝ *α*, and thus also *c*_1_ ∝ *α*, which is subleading compared to the forward trajectories at *θ* ≈ 0, which contribute to the current at order ${{{{{{{\mathcal{O}}}}}}}}(\sqrt{\alpha })$^38^. In contrast, for *Q* > 0, we find that ${c}^{\prime{\prime}}(\pi /2)\propto \alpha \log {\alpha }^{-1}$. The weight of trajectories with steep angles is therefore substantially increased compared to the forward trajectories, which are in turn suppressed and contribute to the current only at order ${{{{{{{\mathcal{O}}}}}}}}(\alpha )$. For small values of *α*, the enhancement of the flat part of the distribution function compared to the forward (ballistic) trajectories is therefore large enough so that a para-hydrodynamic regime emerges (cf. Fig. 1).

Since the logarithmic enhancement makes it impossible to construct the size of *c*_1_ from local properties around *θ* = *π*/2, we now focus on the limit where *α* → 0. To leading order in *α*, in terms of the variable $s=\sin \theta$, Eq. (5) can be expanded as

8 $$\begin{array}{ll}&{s}_{\perp }^{2}\left(2+2Q{s}^{2}-qs\right)\alpha={s}_{\perp }^{2}\left[(2+2Q{s}^{2})\alpha+q{s}^{2}\right]c(s)\\ &-\frac{Q{s}^{2}}{{s}_{\perp }^{2}}\left[s\left(3{s}^{4}-4{s}^{2}+2\right)+2\alpha {s}_{\perp }^{4}\right]{c}^{{\prime} }(s)-Q{s}^{3}{c}^{\prime{\prime}}(s)\end{array}$$

where ${s}_{\perp }=\sqrt{1-{s}^{2}}$. This equation no longer contains any essential singularities for shallow *θ* and is much easier to handle numerically. Based on the analytical structure of *c*^*″*^(*π*/2) in terms of higher derivatives and using the numerical solution as a reference, we find by fitting that ${c}^{\prime{\prime}}(\pi /2)\,\approx \,\alpha \log (0.06/\alpha )/(0.4q+0.5Q)$.

### Effective mean free paths

The crossover from ballistic to bulk hydrodynamic flow is well studied in two-dimensional electron fluids^2,22,42,44–49^ and is typically described using a dual relaxation time approximation for the scattering integral in terms of the two length scales *ℓ*_*mr*_ and *ℓ*_*mc*_. However, it has been shown^44,50^ that the nature of the transport regime can also be reconstructed from an inspection of the distribution function. Namely, the distribution is smooth and relatively flat in the hydrodynamic regime (*ℓ*_*mc*_ ≪ *w* ≪ *ℓ*_*mr*_), while in the ballistic regime (*w* ≪ *ℓ*_*mr*_, *ℓ*_*mc*_), scattering from the boundaries makes the distribution function strongly angle dependent. This can be more formally restated by considering the angular harmonics of *h*(*θ*). Keeping only the first and second terms in such an expansion, the Boltzmann equation simplifies to the Stokes–Ohm equation^44^, i.e., the distribution function can be obtained in a hydrodynamic description. On the other hand, if higher angular harmonics are present in the distribution function, this indicates the presence of additional long-lived modes in the flow, as would be expected for a ballistic distribution function.

In the present case, different parts of the distribution function at respectively shallow or steep angles resemble either the ballistic or the hydrodynamic situation. We therefore propose a two-fluid approximation (cf. Figs. 1 and 3), whereby we decompose the distribution function *h*(*θ*) into a constant part which constitutes a hydrodynamic current density *j*_*h*_ = *c*_1_*j*_0_, while the remaining strongly angle-dependent parts constitute a ballistic current density *j*_*b*_ = *j* − *j*_*h*_.

As shown in Fig. 3b, at *Q* = 0, it is *j*_*h*_ < *j*_*b*_, but upon increasing *Q*, there is a crossover into a regime with *j*_*h*_ > *j*_*b*_. In the latter regime, one can immediately infer *ℓ*_*mr*_ and *ℓ*_*mc*_ from the flat part of the distribution function, which creates the dominant contribution *j*_*h*_ to the current density. To this end, using the reduced current density, we write for the effective momentum-relaxing mean free path,

9 $$\frac{\ell {\ell }_{mr}}{(\ell+{\ell }_{mr})}=\frac{\ell }{\pi }\int\nolimits_{-\pi }^{\pi }{\cos }^{2}\theta c(\theta )\,\approx \,\ell {c}_{1}.$$

The flatness of the distribution function furthermore suggests that the total scattering rate is large and completely dominated by momentum-conserving processes. *ℓ*_*mc*_ is therefore expected to approach its maximally possible value, which for a flat distribution is entirely determined geometrically by the normalized travel distance between two successive scatterings from the top and bottom surfaces. In other words, we can estimate that $\frac{1}{{\ell }_{mc}}\approx \frac{1}{d}\frac{1}{2\pi }d\theta \int\nolimits_{0}^{\pi }\sin|\theta|$, which yields *ℓ*_*mc*_ = *πd*.

Using these values for *ℓ*_*mr*_ ≪ *ℓ* and *ℓ*_*mc*_ ≪ *ℓ*, the effective Gurzhi parameter for the para-hydrodynamic flow in thin sheets becomes

10 $${D}^{{\prime} }=\frac{1}{2}\sqrt{\frac{\ell {\ell }_{mr}}{\ell+{\ell }_{mr}}\frac{\ell {\ell }_{mc}}{\ell+{\ell }_{mc}}}\,\approx \,\frac{\sqrt{\pi }}{2}\sqrt{{c}_{1}d\ell }.$$

In the experiment, the WTe_2_ samples had the properties *d* = 48 nm, *ℓ* = 20 μm, *ℓ*_*mr*_ = 530 nm, and *D* = 155 nm. Using as input the experimental values for *d* and *ℓ*, Eq. (10) yields ${D}^{{\prime} }=145{{{{{{{\rm{nm}}}}}}}}$ and *ℓ*_*mr*_ = 560 nm for the choice (*q*, *Q*) = (1, 4), while it is ${D}^{{\prime} }=143{{{{{{{\rm{nm}}}}}}}}$ and *ℓ*_*mr*_ = 545 nm upon choosing (*q*, *Q*) = (0.8, 2). This indicates that the kinetic theory is not strongly sensitive to the precise value of *q* and *Q*, and it can explain the experimental findings well for a reasonable range of boundary scattering parameters. Importantly, this versatility implies that para-hydrodynamic flow emerges generically as long as there is a large-scale separation between sheet thickness *d* and bulk mean free path *ℓ* and does not require extensive fine-tuning. We remark that *q* and *Q* are, in turn, only weakly dependent on the microscopic scattering parameters *a* and *b*. For example, using *k*_*F*_ = 1 nm^−1^, we obtain *a* = 0.27 nm, *b* = 0.37 nm for (*q*, *Q*) = (1, 4), and *a* = 0.26 nm, *b* = 0.51 nm for (*q*, *Q*) = (0.8, 2). These estimates correspond to boundary roughness at the lattice scale and are indeed reasonable for cleaved samples^34^.

## Discussion

We derived a microscopic boundary scattering model which can explain para-hydrodynamic flow in the absence of strong electron–electron scattering. As the main characteristics of the new regime, we identified singular points in the resulting boundary condition, which lead to a different thickness dependence of the current density that onsets in the presence of small-angle scattering from the boundary. Our findings constitute a new type of ballistic-to-hydrodynamic crossover, where the three-dimensional problem is microscopically ballistic, but the in-plane components of the flow velocity exhibit relaxation properties which are indistinguishable from viscous flows. Our results indicate that the phenomenology which was previously suggested to govern the hydrodynamic-to-ballistic crossover is not universal. Since para-hydrodynamic flow exclusively emerges in the presence of angular diffusion from short-range correlated disorder, conventional approaches which rely solely on a reflectivity coefficient have not been able to capture this mechanism^2,51,52^. We note that small-angle scattering can also appear from bulk scattering, in which case it typically leads to a ratio *ℓ*_*mr*_/*ℓ*_*mc*_ = 4^29,53^. For small-angle boundary scattering, we instead found *ℓ*_*mr*_ = *c*_1_*ℓ* and *ℓ*_*mc*_ = *πd*, which yields a scale-dependent ratio ${\ell }_{mr}/{\ell }_{mc}\propto \log (\ell /d)$ that becomes large enough to support hydrodynamic phenomena for very large ratios *ℓ*/*d*.

It would be interesting to find additional signatures of the para-hydrodynamic regime for the channel flow. Since the in-plane current density in narrow channels is not suitable for distinguishing between the ballistic and hydrodynamic transport regimes^14^, this would probably involve the investigation of the Hall viscosity at finite magnetic fields^46,54^ or optical probes.

Since only very few microscopic parameters other than the bulk mean free path enter into our results, we expect that the para-hydrodynamic flow observed in WTe_2_ is not unique and that a number of clean, three-dimensional compounds, for example, Weyl and Dirac semimetals^4,5^ and ultrapure delafossites^7,55^, can exhibit this new transport regime. The proposed boundary scattering model and the two-fluid approximation used here for extracting the effective mean free paths are generic. Thus, the same methodology can very likely be applied directly to many other three-dimensional materials and also integrated into numerical schemes^56^.

## Methods

The calculation of the electron–electron scattering rate was done in four steps. The first step in the process is finding the energy bands and wave functions of the Weyl semimetal phase of WTe_2_ within DFT using VASP and Wannier90^57,58^. These were evaluated inside the Brillouin zone on a k-mesh of *N*_*x*_ × *N*_*y*_ × *N*_*z*_ = 100 × 50 × 7. The particle-hole bubble Π was calculated with an IR cutoff of 5 meV, which is approximately a tenth of the Fermi energy. The real and imaginary parts were calculated separately to avoid numerical errors. ${{{{{{{\rm{Re}}}}}}}}\,{{\Pi }}(q,\omega )$ and ${{{{{{{\rm{Im}}}}}}}}\,{{\Pi }}(q,\omega )$ were evaluated on the same k-mesh as the bands and wave functions and on energies between −11 and 11 eV with a resolution of 0.055 eV. On energies in between the grid points, a linear interpolation was used. The UV energy cutoff is chosen such that the low-temperature self-energy corrections are converged, where the main contribution to the relaxation time is by electrons with energy *ϵ*_*nq*_ in band *n* residing in an energy window of *ϵ*_*F*_ − *T* < *ϵ*_*nq*_ < *ϵ*_*F*_ + *T* that are scattered by electrons in band *m* that likewise satisfy *ϵ*_*F*_ − *T* < *ϵ*_*mk*_ < *ϵ*_*F*_ + *T*. Thus, the main contribution to the self-energy is generated by electrons whose differences in energies are *ω* = *ϵ*_*nq*_ − *ϵ*_*mk*_ < 2*T*, while the target temperatures are below *T* < 0.025 eV ≪ 11 eV. It is then sufficient to let the band indices run through the first 10 bands above and below the Fermi surface, which corresponds to a maximal energy difference of 4 eV.

The self-energy was calculated with a regularized Bose distribution function, $b(\epsilon )\to ({e}^{\beta \epsilon }-1)/({({e}^{\beta \epsilon }-1)}^{2}+\lambda )$ (cf. Supplementary Information). A good choice for the regulator is *λ* = 10^−4^, such that the pole at zero frequency is cut off at *b*(*ϵ*) < 10^4^.

We confirmed the convergence of our results by comparing various grid resolutions. First, two k-meshes were chosen at sizes 200 × 50 × 3 and 100 × 100 × 3. The difference in the self-energy between both grid resolutions is less than 2%. Additionally, the frequency resolution was also tested by increasing the resolution to 0.02 eV, which yielded a change of less than 1%. Finally, the bosonic occupation number cutoff was increased to *λ* = 10^−^^6^, which yielded a change of less than 1%. The sensitivity of our results against small changes in the Fermi level was also tested. On two points separated by 0.02 eV, the results changed by about 36%, which is significant but shows that the order of magnitude is robust to small changes in the chemical potential. A calculation with a different number of bands included in the correction of the photon propagator showed no relevant changes.

## Supplementary information

Supplementary Information

Peer Review File

## Data Availability

The data that support the findings of this study are available from the corresponding author upon request.
