# Supplementary material for: Para-hydrodynamics from weak surface scattering in ultraclean thin flakes
Source: Nat Commun. 2023 Apr 22;14:2334. doi: 10.1038/s41467-023-37966-z (PMC10122658; doi:10.1038/s41467-023-37966-z)
Supplement: Supplementary file 1 — Supplementary Information [file 41467_2023_37966_MOESM1_ESM.pdf]

Supplementary Information: Para-hydrodynamics from weak surface scattering in ultraclean thin flakes

Yotam Wolf,[1] Amit Aharon-Steinberg,[1] Binghai Yan,[1] and Tobias Holder[1,*]

[1] *Department of Condensed Matter Physics, Weizmann Institute of Science, Rehovot, Israel*

(Dated: March 10, 2023)

In this supplementary information we present the calculation of the electron-electron mean free path, derive the operator of angular diffusion, and detail the solution of the differential equation for boundary scattering.

ELECTRON-ELECTRON COLLISION RATE FROM THE SCREENED COULOMB INTERACTION

To quantify the strength of the interaction we focus the study on the relaxation length, l_{ee}, the mean free path for electron-electron collisions. In Fermi liquid theory normal metals have a relaxation length with a T^{-2} temperature dependence. We emphasize that in WTe_2, the assumption of a T^{-2}-dependence is not justified *a priori*. Instead, it is necessary to calculate l_{ee} down to low temperatures in order to extract a reliable temperature dependence. In our previous calculation [1], we indeed found that the low temperature limit approaches a T^{-2} behavior, but such happens only at energies much smaller than the Fermi level, which in WTe_2 is unusually low at $E_F \approx 150$K. Since the Fermi energy, and the Fermiology of the Fermi surface play a prominent role in WTe_2 [2], and since our previous estimate for $\ell_{ee} \approx 10\mu$m was only 1.5 orders of magnitude to large to explain the hydrodynamic electron flow, here we repeat our calculation for two other band structure candidates for WTe_2. This way, we can make sure that the reported absence of a sufficiently small ℓ_{ee} is not an artifact of the specific choice of band structure, but holds invariably for this material.

Formalism

Electron-electron interactions arise from the coupling of the electrons to the electromagnetic field. In 3D, the bare coulomb interaction is given by $U_{bare}(q) = \frac{4\pi e^2}{|q|^2}$. The presence of electrons modifies the bare interaction, such that the renormalized interaction W becomes

$$U(q,\omega) = \frac{4\pi e^2}{|q|^2 - 4\pi e^2 \Pi(q,\omega)}. \tag{S1}$$

Here, $\Pi(q,\omega)$ is the particle-hole susceptibility of the electromagnetic field, given by the one-loop integral

$$\Pi(q,\omega) = \sum_{i,j} \int \frac{d^3k}{(2\pi)^3} |\rho_{i,k,j,k+q}|^2 \frac{n(\xi_{i,k}) - n(\xi_{j,k+q})}{\xi_{i,k} - \xi_{j,k+q} + \omega - i\eta}. \tag{S2}$$

i, j are band indices, $n(\epsilon)$ is the Fermionic occupation number, $\xi = \epsilon - \epsilon_F$ and $|\rho_{i,k,j,k+q}|^2 = |\langle u_{i,k} e^{iqr} | u_{j,k+q}\rangle|^2$ is the exchange density. At finite temperature the electronic self-energy due to the Fock diagram is

$$\mathrm{Im}\,\Sigma(q,\epsilon_{nq}) = -\pi \sum_m \int \frac{d^3k}{(2\pi)^3} |\rho_{n,q,m,k}|^2 (b(\epsilon_{mk} - \epsilon_{nq}) + n(\epsilon_{mk}))\mathrm{Im}\,U(k-q, \epsilon_{mk} - \epsilon_{nq}), \tag{S3}$$

where $b(\epsilon)$ is the bosonic occupation number. The electron-electron relaxation rate of a state is defined in terms of the self energy as

$$[\tau_{ee}(q,\epsilon_{nq})]^{-1} = \frac{2}{\hbar} \mathrm{Im}\,\Sigma(q,\epsilon_{nq}). \tag{S4}$$

The average relaxation rate is then the average of the relaxation rate on the Fermi surface, normalized by the total density of states:

$$\langle \tau_{ee}^{-1} \rangle_{FS} = \frac{\sum_n \int \frac{d^3k}{(2\pi)^3} [\tau_{ee}(q,\epsilon_{nq})]^{-1} \frac{\partial f_{nq}}{\partial \epsilon_{nq}}}{\sum_n \int \frac{d^3k}{(2\pi)^3} \frac{\partial f_{nq}}{\partial \epsilon_{nq}}} \tag{S5}$$

Finally, the relaxation length is given as the inverse of the relaxation rate,

$$l_{ee} = v_F \langle \tau_{ee}^{-1} \rangle_{FS}^{-1}. \tag{S6}$$

Candidate band structures

Weyl semimetal candidate band structure The chemical potential resides at $\mu = 7.22eV$, the lattice parameters are $a_x = 0.3483nm, a_y = 0.6265nm, a_z = 1.4043nm$. The resulting parameters are listed in Supplementary Table I.

n	$\epsilon_f[eV]$	$v_F[10^5 m/s]$	$(k_F)_x[nm^{-1}]$	$(k_F)_y[nm^{-1}]$	$(k_F)_z[nm^{-1}]$	$g(\epsilon_f)[eV^{-1}nm^{-3}]$	$n(\epsilon_f)[nm^{-3}]$
55	0.0496	1.09	0.60	0.63	0.54	2.1655	0.0631
56	0.0834	1.327	0.61	0.92	0.54	2.9942	0.1056
57	0.0691	2.35	0.34	0.60	0.93	1.0546	0.0498
58	0.0679	2.32	0.30	0.55	0.89	0.9026	0.0393

Supplementary Table I. Details of the Weyl semimetal phase.

Relaxed candidate band structure The chemical potential sits at $\mu = 8.5083$. This band structure is motivated by Ref. [2], with lattice parameters $a_x = 0.3460nm, a_y = 0.6200nm, a_z = 1.3090nm$. Compared to the other band structures, this corresponds to a compression along the k_z-direction. The position of the atoms inside the lattice was subsequently optimized to minimize the total energy. The result is shown in Supplementary Table II.

n	$\epsilon_f[eV]$	$v_F[10^5 m/s]$	$(k_F)_x[nm^{-1}]$	$(k_F)_y[nm^{-1}]$	$(k_F)_z[nm^{-1}]$	$g(\epsilon_f)[eV^{-1}nm^{-3}]$	$n(\epsilon_f)[nm^{-3}]$
55	0.1944	2.91	1.23	1.48	1.27	1.4147	0.2752
56	0.2074	2.95	1.35	1.74	1.28	1.7539	0.3501
57	0.2565	2.80	0.73	1.48	1.11	2.6862	0.3231
58	0.2307	3.03	0.64	1.30	1.15	1.9589	0.2338

Supplementary Table II. Details of the relaxed band structure.

Candidate band structure based on hybrid functionals Here, the chemical potential is $\mu = 6.965eV$. The chosen lattice parameters are $a_x = 0.3483nm, a_y = 0.6265nm, a_z = 1.4043nm$. Supplementary Table III lists the band structure parameters obtained when using hybrid functionals in the self consistent stage of the calculation in order to better account for exchange.

n	$\epsilon_f[eV]$	$v_F[10^5 m/s]$	$(k_F)_x[nm^{-1}]$	$(k_F)_y[nm^{-1}]$	$(k_F)_z[nm^{-1}]$	$g(\epsilon_f)[eV^{-1}nm^{-3}]$	$n(\epsilon_f)[nm^{-3}]$
55	0.0669	2.57	-	-	-	0.2158	0.0065
56	0.0801	2.49	-	-	-	0.2923	0.0101
57	0.0430	2.95	-	-	-	0.3450	0.0120
58	0.0419	2.68	-	-	-	0.1587	0.0040

Supplementary Table III. Details of the band structure obtained from hybrid functionals.

We briefly comment on consistency checks using the density of states, and why they are less relevant in the present calculation. For example, the total density of states of the relaxed phase is $7.81eV^{-1}nm^{-3}$ and according to the calculation of the real part of the susceptibility at $\omega = 0.01eV$ it is $6.47eV^{-1}nm^{-3}$, which agrees within 20% with the total density of states. Likewise, the total density of states of the Weyl semimetal phase is $7.12eV^{-1}nm^{-3}$ and according to the calculation of the real part of the susceptibility at $\omega = 0.01eV$ it is $4.27eV^{-1}nm^{-3}$, which is further apart, with a 67% difference. As the main reason we suspect the more sensitive dependence on ω in the Weyl semimetal band structure. However, for a faithful estimate of the self-energy this effect is not relevant because we are predominantly interested in the imaginary part of U, which becomes zero exactly at $\omega = 0$.

Results for the electron-electron mean free path

The electron-electron mean free path according to Supplementary Eq. (S6) and its temperature scaling are shown in Supplementary Fig. 1 for all three candidate band structures. Clearly, the low-energy asymptotic scaling onsets at or even slightly above 150K in all three cases. Extrapolating the obtained temperature scaling, we arrive at the low-temperature quoted in the main text.

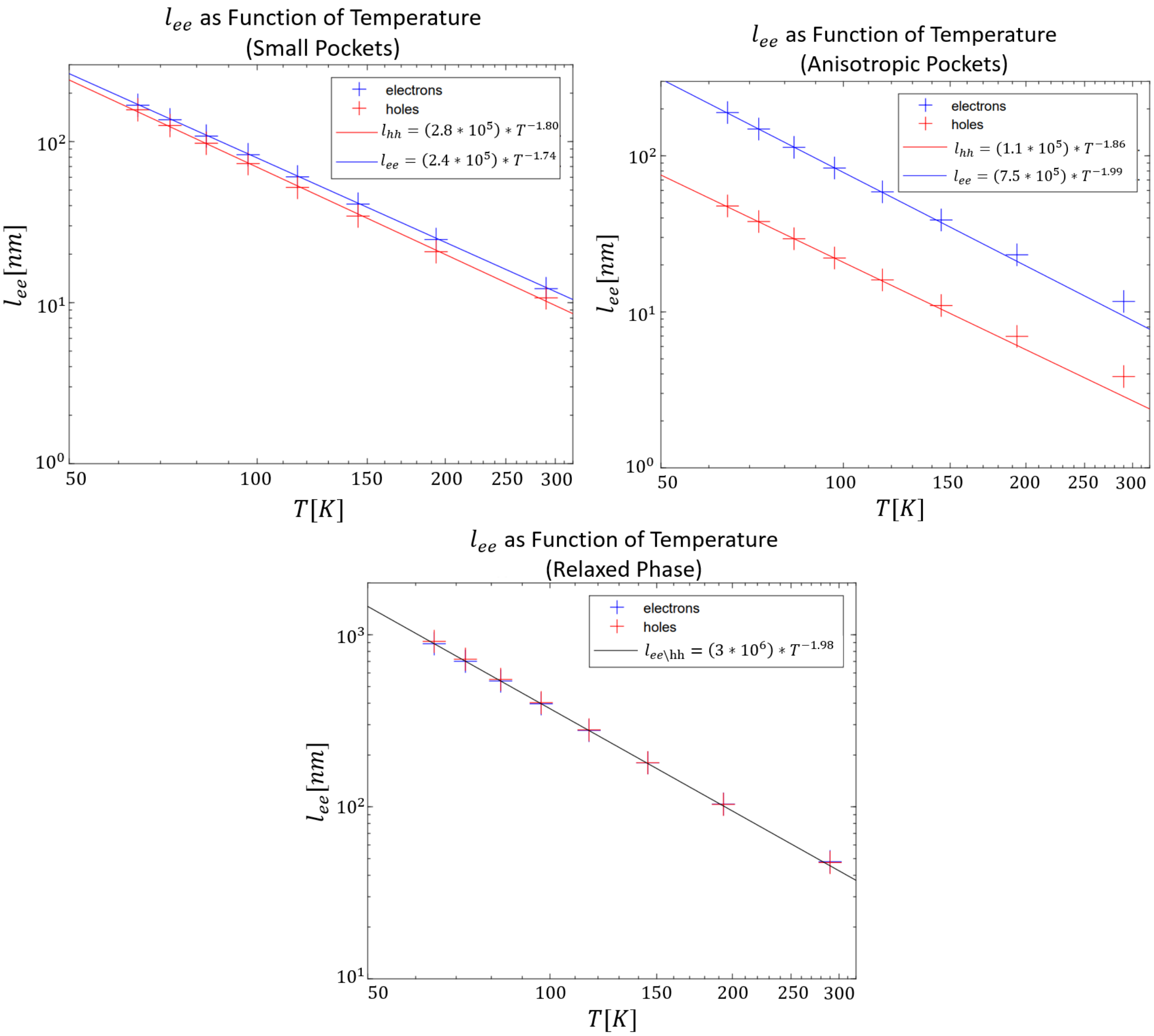

Supplementary Figure 1. l_{ee} as function of temperature for the three band structures. (a) Small pockets: due to the compensation of electrons and holes and the similar velocities of the bands, l_{ee} and l_{hh} are similar, we can see a good fit to a power law, but with a weaker dependence on temperature than inverse square. (b) Anisotropic pockets: the Fermi level was chosen in a way that the compensation of holes and electrons is not perfect, more electrons than holes. This is reflected both in an effective lower Fermi level and lower velocity for the holes, which manifest in the form of $l_{hh} < l_{ee}$. Still, we can see a good fit to a power law, specifically for electrons, a T^{-2}. (b) Relaxed phase: resembels a metal and as such fits well to a T^{-2} power law. The relatively large l_{ee} compared to the previous two band structures is mainly due to the large Fermi level, which leads to high screening, reducing the effective interaction.

DIFFERENTIAL OPERATOR

The derivation of the differential operator was first done in Ref. [3], but uses a highly customized notation. For this reason, we reiterate here the steps needed to obtain $\hat{O}_\theta$. We start from the general boundary scattering condition in terms of the scattering potential (Eq. (2) in the main text), $f^>(\boldsymbol{k}) = f^<(\boldsymbol{k}) + I$ where the integral is defined as

$$I = k_z \int k'_z (f^<(\boldsymbol{k}') - f^<(\boldsymbol{k})) W(\boldsymbol{k}', \boldsymbol{k}) \tag{S7}$$

Recalling that the scattering potential has the explicit form $W(\boldsymbol{k}', \boldsymbol{k}) = W_0 e^{-\frac{|\boldsymbol{k}-\boldsymbol{k}'|^2}{2\sigma^2}}$, we proceed with a saddle point approximation. Denoting $g(\boldsymbol{k}') = k'_z(f^<(\boldsymbol{k}') - f^<(\boldsymbol{k}))$, it is clear that $g(\boldsymbol{k}) = 0$. Expanding up to second order in

$\boldsymbol{k}' - \boldsymbol{k}$, in spherical coordinates on the Fermi surface, it is therefore

$$g(\boldsymbol{k}') = \sum_i [\partial_i g(\boldsymbol{k})](k' - k)_i + \frac{1}{2} \sum_{ij} [\partial_i \partial_j g(\boldsymbol{k})](k' - k)_i (k' - k)_j \tag{S8}$$

Inserting the expansion into the integral $I = I_1 + I_2$, we obtain

$$I_1 = W_0 k_z \sum_i [\partial_i g(\boldsymbol{k})] \int (k' - k)_i e^{-\frac{|\boldsymbol{k}-\boldsymbol{k}'|^2}{2\sigma^2}} \tag{S9}$$

$$I_2 = \frac{1}{2} k_z W_0 \sum_{i,j} [\partial_i^2 g(\boldsymbol{k})] \left[\int (k' - k)_i (k' - k)_j e^{-\frac{|\boldsymbol{k}'-\boldsymbol{k}|^2}{2\sigma^2}} \right] \tag{S10}$$

In polar coordinates relative to $\boldsymbol{k}$, we notice that $(k' - k)_i$ is anti-symmetric with respect to the transformation $\boldsymbol{k}' \to 2\boldsymbol{k} - \boldsymbol{k}'$, while $e^{-\frac{|\boldsymbol{k}-\boldsymbol{k}'|^2}{2\sigma^2}}$ is symmetric. Therefore the integral on the components that are perpendicular to $\boldsymbol{k}$, meaning $i = \theta, \varphi$ vanish. On the other hand, the integral on the radial direction, does not vanish from this argument, but instead vanishes identically, as $|\boldsymbol{k}'| = |\boldsymbol{k}|$ is enforced identically on the entire integration range. Thus it is $I_1 = 0$.

For I_2, obviously the same argument holds for the radial direction. It thus suffices to look at directions i, j that are perpendicular to $\boldsymbol{k}$. Considering $i \neq j$ next, the integrand is odd in either direction and vanishes due to antisymmetric cancellation. So the only terms that contribute are $i = j$ for the two directions perpendicular to $\boldsymbol{k}$,

$$I_2 = \frac{1}{2} p_z W_0 \sum_{i=1,2} [\partial_i^2 g(\boldsymbol{k})] \left[\int (k' - k)_i^2 e^{-\frac{|\boldsymbol{k}'-\boldsymbol{k}|^2}{2\sigma^2}} \right].$$

The integral inside the brackets can be solved by noticing that for the two directions perpendicular to $\boldsymbol{k}$, the result is the same from rotational symmetry around the $\boldsymbol{k}/|\boldsymbol{k}|$ axis:

$$\int (k' - k)_i^2 e^{-\frac{|\boldsymbol{k}'-\boldsymbol{k}|^2}{2\sigma^2}} = \frac{1}{2} \int (\boldsymbol{k}' - \boldsymbol{k})^2 e^{-\frac{|\boldsymbol{k}'-\boldsymbol{k}|^2}{2\sigma^2}} = -\sigma^2 k_F^2 \frac{\partial}{\partial \alpha} \left[e^{-\frac{\alpha k_F^2}{\sigma^2}} \int e^{\frac{\alpha k_F^2 \cos\theta}{\sigma^2}} \sin\theta d\theta d\varphi \right] |_{\alpha=1} \tag{S11}$$

$$= 2\pi\sigma^4 \left[\frac{2k_F^2}{\sigma^2} e^{-\frac{2k_F^2}{\sigma^2}} - (1 - e^{-\frac{2k_F^2}{\sigma^2}}) \right] \equiv \frac{2Q}{W_0} \tag{S12}$$

In summary, the integral I evaluates to

$$I = I_2 = k_z \sum_{i=1,2} [\partial_i^2 g(\boldsymbol{k})] Q = Q k_z \Big[\sum_{i=1,2} \partial_i^2 \Big] (k_z f^<(\boldsymbol{k})). \tag{S13}$$

We note that ∂_i^2 where $i = \theta, \phi$ is exactly the Laplace-Beltrami operator on the sphere. We can therefore write more formally

$$f^>(\boldsymbol{k}) = f^<(\boldsymbol{k}) + Q k_z \nabla^2 (k_z f^<(\boldsymbol{k})). \tag{S14}$$

Using the identity $\nabla^2(k_z f^<(\boldsymbol{k})) = f^<(\boldsymbol{k}) \nabla^2 k_z + k_z \nabla^2 f^<(\boldsymbol{k}) + 2\nabla k_z \cdot \nabla f^<(\boldsymbol{k})$ and noting that $\nabla^2 \boldsymbol{k}_z = 0$, we recover the form of Ref. [3],

$$f^>(\boldsymbol{k}) = \Big(1 + Q k_z (\nabla k_z \cdot \nabla + 2 k_z \nabla^2) \Big) f^<(\boldsymbol{k}). \tag{S15}$$

In spherical coordinates, this is

$$\nabla k_z \cdot \nabla = (-\sin\theta \hat{\theta}) \cdot (\hat{\theta} \frac{1}{k_F} \partial_\theta + \hat{\varphi} \frac{1}{k_F \sin\theta} \partial_\varphi) = -\frac{\sin\theta}{k_F} \partial_\theta \tag{S16}$$

$$\nabla^2 = \frac{1}{k_F^2 \sin\theta} \partial_\theta (\sin\theta \partial_\theta) + \frac{1}{k_F^2 \sin^2\theta} \partial_\varphi^2 \tag{S17}$$

Since we consider a thin slab where the thickness d is much smaller than the width w, we can neglect derivatives with respect to the in-plane angle ϕ, which are expected to only contribute weakly due to the smooth dependence of f on ϕ. In term of the reduced distribution function $h(z, \theta)$ the boundary condition therefore simplifies to

$$h(d/2, -|\theta|) = \Big[1 + Q \cos\theta \big(\cot\theta \partial_\theta (\sin\theta \partial_\theta) - 2 \sin\theta \partial_\theta \big) \Big] h(d/2, |\theta|) \tag{S18}$$

$$= \Big[1 + Q \cos^2\theta \big((\cot\theta - 2\tan\theta) \partial_\theta + \partial_\theta^2 \big) \Big] h(d/2, |\theta|), \tag{S19}$$

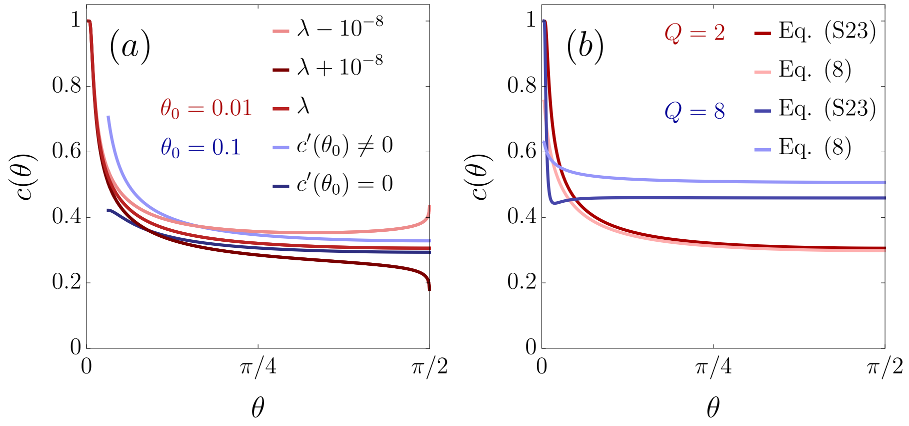

Supplementary Figure 2. (a) Iterative solution of the differential equation (S20). Parameters are $\alpha = 0.05$, $Q = 2$, $\theta_0 = 0.2\alpha$. The converged solution is obtained for $\lambda = 0.0098715246968$, and the solution is very sensitive to the initial conditions, deviating substantially already when changing λ by only 10^{-8}. In blue we show less precise solutions which have been obtained for a much larger θ_0, for two different choices for $c'(\theta_0)$. Nevertheless, their deviation from the correct value at $\theta = \pi/2$ remains small. (b) Comparison between the exact differential equation (S20), and the expansion for small α (Eq. (8) of the main text), for a comparatively sizable $\alpha = 0.05$. Both compare favorably for moderate values of Q. Only if $Q \gg 1$, the solutions deviate noticeably. In all examples the specularity parameter is $q = 1$.

which, after shifting $\theta \to \theta - \pi/2$ and adding the specularity coefficient R_θ is the form quoted in the main text in Eq. (3). While it may look as though Supplementary Eq. (S19) contains a divergence at at $\theta = 0$, we point out that this divergence is of the form $1/\sin\theta$, which is rendered finite upon integration with the Jacobian $k_F^2 \sin\theta$ on the sphere.

SOLUTION OF THE DIFFERENTIAL EQUATION FOR BOUNDARY SCATTERING

The differential boundary condition, Eq. (5) in the main text, can be rewritten in terms of the variable $s = \sin\theta$, and reads explicitly

$$\begin{aligned}
&\left(1-s^2\right)\left[\alpha^2 Q - s^3\left(q + 2\alpha Q - q e^{\alpha/s}\right) + s^2\left(1 - \alpha^2 Q - e^{\frac{2\alpha}{s}}\right)\right] \\
&= \left(1-s^2\right)\left[\alpha^2 Q - s^3(q + 2\alpha Q) + s^2\left(1 - \alpha^2 Q - e^{\frac{2\alpha}{s}} + 1\right)\right] c(s) \\
&+ \frac{Q s^2}{1-s^2}\left[2\alpha\left(1-s^2\right)^2 + s\left(3s^4 - 4s^2 + 2\right)\right] c'(s) + Q s^4 c''(s).
\end{aligned} \tag{S20}$$

A unique solution for $c(\theta)$ is obtained by imposing smooth continuity for this periodic and even function, i. e. we demand that $c'(0) = c'(\pi/2) = 0$. However, since $\theta = 0, \pi/2$ are singular points, this way of solving Supplementary Eq. (S20) is impractical, and it is preferable to instead use initial conditions for $c(\theta_0)$ and $c'(\theta_0)$, where $\theta_0 \approx 0$ is chosen small enough for the effect of the derivatives in Supplementary Eq. (S20) to become vanishingly small at θ_0. This makes sense because for $\theta \to 0$ Eq. (5) of the main text can be solved algebraically, yielding

$$c_0(\theta) = 1 - \frac{q e^{\alpha \csc\theta}}{q + \csc\theta\left(e^{2\alpha\csc\theta} - \alpha^2 Q \cot^2\theta - 1\right) + 2\alpha Q}. \tag{S21}$$

Then, using initial values $c(\theta_0) = 1 - (1+\lambda)(1 - c_0(\theta))$ and $c'(\theta_0) = (1+\lambda)c_0'(\theta)$, and slowly adjusting the tuning parameter $\lambda \approx 0$ such that the solution does not diverge at $\pi/2$ presents a reliable way to construct physically relevant solutions for $c(\theta)$. A typical iterative solution is presented in Supplementary Fig. 2

Unfortunately, this procedure still encounters issues at small α as the distribution function becomes exponentially close to 1, which means that the initial condition $c(\theta_0) \approx 1$ is rendered increasingly sensitive to machine-precision limitations. We found two avenues to remedy this issue.

One approach amounts to choosing a much larger θ_0, which obviously cannot capture the form of $c(\theta)$ for angles below θ_0, but converges smoothly to the correct solution at large θ. The second option is to expand Supplementary

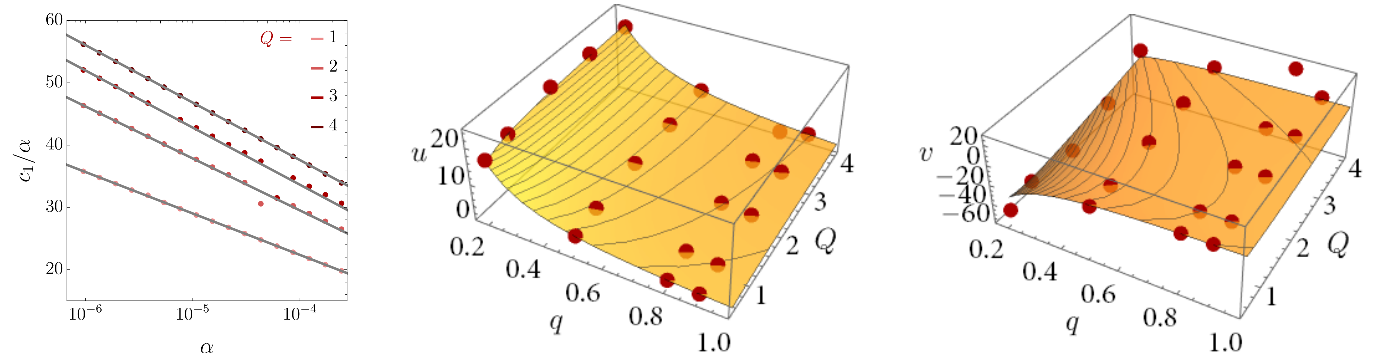

Supplementary Figure 3. (Left) Logarithmic divergence of c_1/α in the limit $\alpha \to 0$, obtained from solving Eq. (8) of the main text, for the parameter $q = 0.9$. The gray curves show the fit with a logarithm, $c_1(\alpha)/\alpha = u \log \alpha^{-1} + v$. (Middle, Right) Results from fitting the relation $c_1(\alpha)/\alpha$ with the parameters u (v) are shown in the middle (right). The red points are values of u and v as determined from numerically solving Eq. (8) as a function of α for various values of (q, Q). Overlaid are the functions Eqs. (S25,S26), for the parameter values $\lambda_2 = 0.39$, $\lambda_3 = 0.46$ and $\lambda_1 = 0.061$.

Eq. (S20) in the limit $\alpha \to 0$. Doing so removes the exponential functions, and thus the essential singularities at $\theta = 0$, so that the choice of very small θ_0 remains unproblematic even for small α. Supplementary Fig. 2 shows the differences and commonalities between both approximations.

We calculate the limiting value $c(\pi/2) \equiv c_1$ in two steps. Using the expanded differential equation, Eq. (8) in the main text, the canonical expansion of $c(\theta)$ around $\pi/2$ yields

$$c(\tfrac{\pi}{2}) = \frac{2Q}{q} c''(\tfrac{\pi}{2}) + \frac{2 - q + 2Q}{q}\alpha \tag{S22}$$

$$c''(\tfrac{\pi}{2}) = \frac{4Q}{3q + 20Q} c^{(4)}(\tfrac{\pi}{2}) + \frac{3(4 - q)}{3q + 20Q}\alpha \tag{S23}$$

$$c^{(4)}(\tfrac{\pi}{2}) = \frac{2Q(3q + 20Q)}{5q^2 + 120qQ + 488Q^2} c^{(6)}(\tfrac{\pi}{2}) + \frac{q(-25q - 234Q + 160) + 1336Q}{5q^2 + 120qQ + 488Q^2}\alpha \tag{S24}$$

with all odd derivatives being equal zero. The expansion indicates that no non-analyticities are acquired at any finite order. On the other hand, as is visible in Supplementary Fig. 3, c_1/α as a function of α reveals clearly contains a logarithmic divergence $c_1 \propto \alpha \log \alpha^{-1}$. Based on the structure displayed in the sequence of derivatives, we therefore make the ansatz that $c''(\pi/2) \approx \alpha \log(\lambda_1/\alpha)/(\lambda_2 q + \lambda_3 Q)$ with three fitting parameters λ_1, λ_2 and λ_3.

Then, we perform a numerical fit to $c_1(\alpha)/\alpha = u \log \alpha^{-1} + v$ for a range of values of (q, Q). The result of this fit is shown in Supplementary Fig. 3. Obviously, the fitting parameters u and v are related to λ_i via

$$u = \frac{2Q}{q} \frac{1}{\lambda_2 q + \lambda_3 Q} \tag{S25}$$

$$v = \frac{2Q \frac{\log(\lambda_1)}{\lambda_2 q + \lambda_3 Q} + (2 - q + 2Q)}{q} \tag{S26}$$

These relations are used to fit $\lambda_2 = 0.39$ and $\lambda_3 = 0.46$ from u in Supplementary Eq. (S25) and after fixing them, to also fit $\lambda_1 = 0.061$ from v, Supplementary Eq. (S26), thus yielding the approximate form of $c''(\pi/2)$ stated in the main text.

* tobias.holder@weizmann.ac.il

[1] A. Aharon-Steinberg, T. Völkl, A. Kaplan, A. K. Pariari, I. Roy, T. Holder, Y. Wolf, A. Y. Meltzer, Y. Myasoedov, M. E. Huber, B. Yan, G. Falkovich, L. S. Levitov, M. Hücker, and E. Zeldov, Nature **607**, 74–80 (2022), arXiv:2202.02798 [cond-mat.mes-hall].

[2] U. Vool, A. Hamo, G. Varnavides, Y. Wang, T. X. Zhou, N. Kumar, Y. Dovzhenko, Z. Qiu, C. A. C. Garcia, A. T. Pierce, J. Gooth, P. Anikeeva, C. Felser, P. Narang, and A. Yacoby, Nature Physics **17**, 1216 (2021), arXiv:2009.04477 [cond-mat.mes-hall].

[3] L. A. Falkovsky, Advances in Physics **32**, 753 (1983).
