# Supplementary material for: Para-hydrodynamics from weak surface scattering in ultraclean thin flakes
Source: Nat Commun. 2023 Apr 22;14:2334. doi: 10.1038/s41467-023-37966-z (PMC10122658; doi:10.1038/s41467-023-37966-z)
Supplement: Supplementary file 2 — Peer Review File [file 41467_2023_37966_MOESM2_ESM.pdf]

nature portfolio

Peer Review File

Para-Hydrodynamics from weak surface scattering in ultraclean thin flakes

Open Access This file is licensed under a Creative Commons Attribution 4.0 International License, which permits use, sharing, adaptation, distribution and reproduction in any medium or format, as long as you give appropriate credit to the original author(s) and the source, provide a link to the Creative Commons license, and indicate if changes were made. In the cases where the authors are anonymous, such as is the case for the reports of anonymous peer reviewers, author attribution should be to 'Anonymous Referee' followed by a clear attribution to the source work. The images or other third party material in this file are included in the article's Creative Commons license, unless indicated otherwise in a credit line to the material. If material is not included in the article's Creative Commons license and your intended use is not permitted by statutory regulation or exceeds the permitted use, you will need to obtain permission directly from the copyright holder. To view a copy of this license, visit http://creativecommons.org/licenses/by/4.0/.

Reviewers' Comments:
Reviewer #2:

Para-Hydrodynamics from weak surface scattering in ultraclean thin flakes

The manuscript titled *"Para-Hydrodynamics from weak surface scattering in ultraclean thin flakes"* investigates the microscopic kinetic origins of an interesting question arising from recent electron-hydrodynamic experiments at low-temperatures, namely: 'does nearly-specular surface scattering from the top and bottom surfaces of a *thin* conductor enhance the *in-plane* momentum-conservation in experimental *signatures*'?

The results appear scientifically rigorous and internally consistent. In particular, the analytical derivation of the 1D channel distribution is very nice, and provides an analytically-simple extension for specular boundary conditions amenable to numerical calculations.

Considering the recent interest in the field of electron hydrodynamics, and the advent of spatially-resolved current density measurements in spatially-complex geometries, this work is very timely and will be a worthwhile contribution to the field.

Main Concerns

I have three main concerns, which pertain to the scope of the claims the manuscript makes given the approximation of neglecting the spatial dependence along the width of the channel:

1. If I understand the experiments (which the manuscript draws motivation from describing) correctly, they measure the projected induced magnetic flux along the width of the channel. As such, I was expecting the work in this manuscript to draw conclusions on the *in-plane current densities*, given the choice of *out-of-plane boundary conditions*.

   This indeed has a natural precedent in ideal fluids, which I believe would be beneficial for the authors to mention: The 'Poiseuille' velocity profile in a rectangular channel is of the form:

$$u(y,z) = \frac{4h^2\Delta p}{\pi^3 \eta L} \sum_{n,odd}^{\infty} \frac{1}{n^3} \left[1 - \frac{\cosh\left(n\pi \frac{y}{h}\right)}{\cosh\left(n\pi \frac{w}{2h}\right)}\right] \sin\left(n\pi \frac{z}{h}\right)$$

   In the regime the manuscript is concerned about ($h/w \to 0$) , the above develops an in-plane current density which deviates from the usual parabolic profile to a 'snubbier' current profile. Note: of-course the above assumes no-slip boundary conditions, and in-general a kinetic theory vs a pde-hydrodynamic solution would be desirable in the case in question (as the authors rightly point out.)

   My confusion stems from the following: "How can the authors conclude about the nature of experimental results measuring in-plane current densities, when their analysis rules them out by assumption?"

2. In a similar vein, the authors use an isotropic Fermi velocity in their analysis, claiming that in-contrast to the highly-anisotropic Fermi surfaces the Fermi velocity is fairly isotropic in a footnote. This is fine, although calculations showing the distribution of the Fermi velocity in the SI would be more convincing. However, since the Fermi velocity enters the BTE as:

$$\boldsymbol{v}_F \cdot \nabla_{\boldsymbol{r}} f + e\boldsymbol{E} \cdot \nabla_{\boldsymbol{k}} f = I[f]$$

then in-order for their assumption pointed out in 1. above to be justified, they would require instead that $v_y \to 0$, not that it's isotropic in 3D. This is clearly violated in WTe_2 looking at Figure 2.

3. The bulk collision integral used in (1), given by the single-relaxation time approximation $I_0[f] = |\boldsymbol{v}|\,(f - f_0)/l$ conserves no collisional invariants and thus cannot be used to construct hydrodynamic theories. This is nicely discussed in eq. (9) and Appendix A of de Jong and Molenkamp (Phys. Rev. B 51, 13389). While this is usually rendered a moot-point due to the symmetry of the channel geometry, the authors need to be more careful in general geometries such as the experimental double-chamber geometry they're referencing. More generally, it would be nice if the authors could include a momentum-conserving term in their bulk collision integral, e.g. using the dual-relaxation time approximation. I understand this will of-course make the analytical solution much harder, so it might be out-of scope.

Minor Points

- I believe the term 'para-hydrodynamics' is unnecessarily confusing, and would urge the authors to remove it. As illustrated above with the velocity profile of an *ideal fluid* in a rectangular channel, the geometry let alone the boundary conditions naturally affect the observed current densities. To the extent that "electron hydrodynamics" is used to describe electron flow that's "fluid-like" the term "hydrodynamics" should be taken to quantify the extent to which momentum is conserved in the system.
- Please include ϕ in your schematic in Figure 1a.
- Please use a different notation than D for your modified Gurzhi parameter in (10). Currently the two sentences immediately after (10) are rather confusing.
- The discussion in the last results section *Effective mean free paths* is a bit too high-level. Please explain how the "nature" of the transport regime can be reconstructed by inspecting the distribution function more clearly (I found myself digging through references [47,53] to grasp this), especially since the distribution function is geometry dependent?
- I believe the divergences in the angular diffusion operator should be discussed in more detail. In particular, approaches to regularize them in numerical implementations (see below) would be useful.

If the authors can adequately alleviate my confusion on the scope of the claims, I would be happy to recommend this for publication in *Nature Communications*.

Proof-of-concept Calculations Guiding Concerns

I tried to resolve some of the confusion discussed above, namely points 1. and 2., by the following proof-of-concept 3D numerical calculations. In case it's useful for the authors, I include them here.

In particular, I investigated a simple channel geometry with the following parameters:

- Slenderness ratio ($h/w = 1/10$)
- *3D* Isotropic Fermi velocity ($N = 256$ carriers distributed evenly on the surface of a unit sphere)
- Three different isotropic bulk collision integrals using the dual-relaxation time approximation:
  - "diffusive": ($l_{mr}/w = 0.05$, $l_{mc}/w = 10$)
  - "hydrodynamic": ($l_{mr}/w = 10$, $l_{mc}/w = 0.05$)
  - "ballistic": ($l_{mr}/w = 10$, $l_{mc}/w = 10$)

- Periodic boundary conditions along the length of the channel
  (i.e. carriers exiting the right of the geometry get injected back on the left, and vice versa)
- Diffusive boundary conditions along the width of the channel
  (i.e. carrier distributions exiting the front of the geometry get uniformly scattered into backward-traveling carriers)
- Three different boundary conditions along the height of the channel
  - Diffusive boundary conditions
    (as above)
  - Specular boundary conditions as in the paper, $R_\theta = 1 - q\sin(|\theta|)$
    ($q = 0.5$)
  - Specular + angular diffusion operator as in the paper,
    $\hat{O}_\theta = Q\sin^2(\theta)\left((2\cot(\theta - \tan(\theta)))\partial_\theta + \partial_\theta^2\right)$
    ($Q = 2$, ∂_θ and ∂_θ^2 computed using central finite differences of order 4)

i) First, note it's easy to show that using the "hydrodynamic" bulk collision integral and diffusive boundary conditions for $h/w = 1/10$ and $h/w = 1$ one indeed obtains 'snubby' and 'parabolic' profiles respectively (as shown analytically above in point 1.). This suggests that point 2. above is important, i.e. that the 3D nature of the Fermi velocity is important, and one needs to justify $v_y \to 0$.

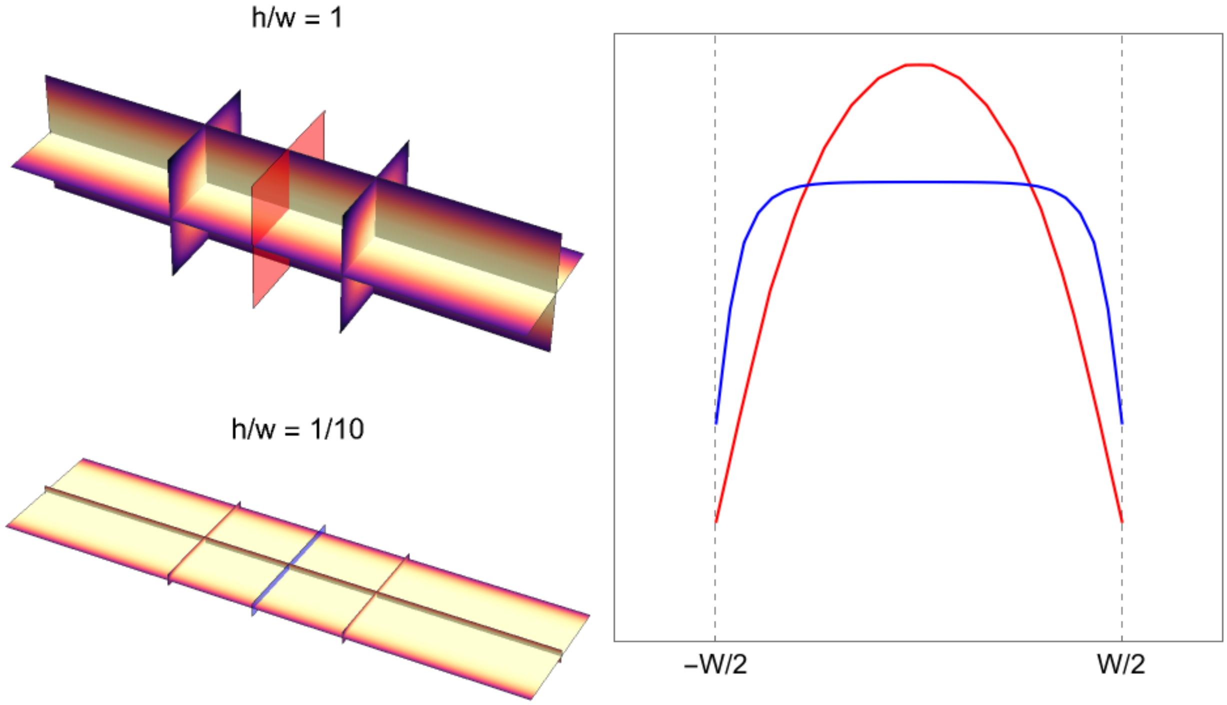

ii) Second, it is indeed *true* that for the slender geometries ($h/w = 1/10$) making the out-of-plane boundary conditions specular ($q = 0.5, Q = 0$) alters the in-plane current distributions. However, the effect is negligible when using "diffusive" bulk collision integrals, small when using "ballistic" bulk collision integrals, and strong when using "hydrodynamic" bulk collision integrals.
This suggests that point 1. above is important and supports the authors claims, however the phenomenon is not unique (and in-fact weaker) in the "ballistic" regime, and did not require the angular-diffusion operator.

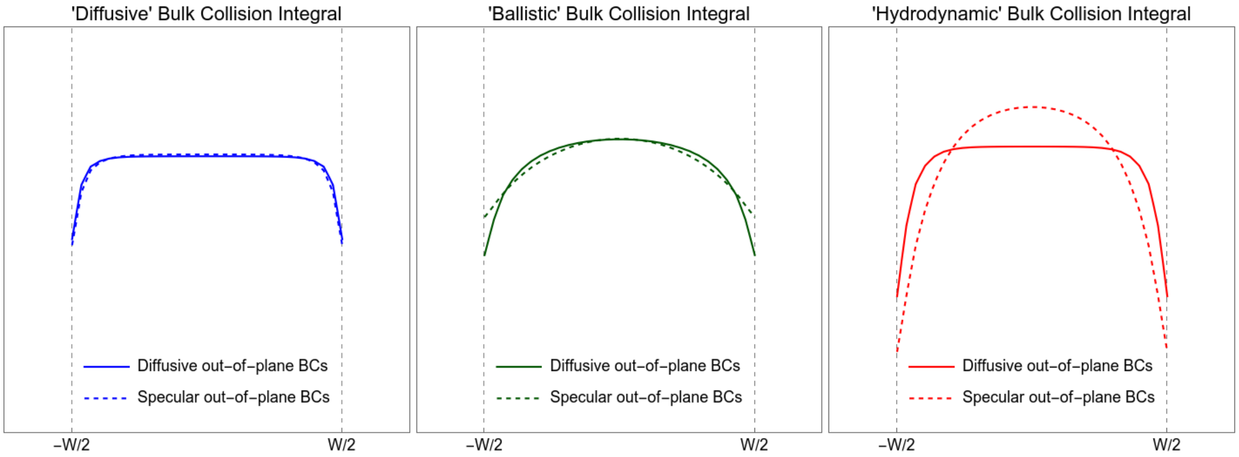

iii) Third, the angular diffusion boundary condition ($q = 0.5, Q = 2$) resulted in numerical instabilities. I believe this is due to the divergences highlighted above? Alternatively, the authors can suggest in the SI a better discretization scheme for the angular derivatives other than central finite differences.

Georgios Varnavides

Referee Report

The manuscript titled "Para-Hydrodynamics from weak surface scattering in ultra-clean thin flakes" investigates the microscopic kinetic origins of an interesting question arising from recent electronhydrodynamic experiments at low-temperatures, namely: 'does nearly-specular surface scattering from the top and bottom surfaces of a thin conductor enhance the in-plane momentum-conservation in experimental signatures'? The results appear scientifically rigorous and internally consistent. In particular, the analytical derivation of the 1D channel distribution is very nice, and provides an analytically-simple extension for specular boundary conditions amenable to numerical calculations. Considering the recent interest in the field of electron hydrodynamics, and the advent of spatially resolved current density measurements in spatially-complex geometries, this work is very timely and will be a worthwhile contribution to the field.

[...]

If the authors can adequately alleviate my confusion on the scope of the claims, I would be happy to recommend this for publication in Nature Communications.

We thank the referee for his positive assessment of the content and impact of our work, and his (tentative) recommendation for publication. We also thank the referee for the great amount of work which he invested in setting up a numerical calculation to check our results. We are highly confident that we can fully address his remaining points of concerns in our response.

Main Concerns

I have three main concerns, which pertain to the scope of the claims the manuscript makes given the approximation of neglecting the spatial dependence along the width of the channel:

The referee is correct that for our calculation we do not consider the spatial dependence of the current density perpendicular to the channel direction. This is a simplification which allowed us to derive our semi-analytical solutions and is central to the approach. However, this approximation does not *imply that $v_y \to 0$, neither does it imply that the resulting (three-dimensional) flow is hydrodynamic, or that hydrodynamic approaches can capture the three-dimensional flow profile. Quite the opposite - the limit that we consider is given by $h/w \to 0$ combined with $\ell_{ee} \to \infty$ and lastly with rather specular in-plane boundaries, i.e. it corresponds to a microscopically ballistic fluid which is disturbed marginally due to surface scattering in the thin flake limit, such that it develops two fluid components which flow uniformly along the width of the channel. The resulting flow is still microscopically ballistic (meaning that it is not possible to use a Stokes-Ohm hydrodynamic description for the three-dimensional distribution function), but the in-plane components of the flow velocity exhibit relaxation properties which are indistinguishable from hydrodynamic (viscous) flows. This is our main result: The effective in-plane momentum conserving and momentum relaxing mean free paths as derived*

from the microscopic distribution function exhibit a logarithmic scale separation. This novel, intermediate regime is therefore unlike neither ballistic nor hydrodynamic flow, and thus presents a unique and new flow regime which is exclusively produced by the dimensional crossover. Importantly, it was not reported before anywhere in the literature. In order to make this point clearer, in the updated manuscript we have expanded the introductory text where we explain our approach.

1. If I understand the experiments (which the manuscript draws motivation from describing) correctly, they measure the projected induced magnetic flux along the width of the channel. As such, I was expecting the work in this manuscript to draw conclusions on the in-plane current densities, given the choice of out-of-plane boundary conditions. This indeed has a natural precedent in ideal fluids, which I believe would be beneficial for the authors to mention: The 'Poiseuille' velocity profile in a rectangular channel is of the form:

$$u(y,z) = \frac{4h^2\Delta p}{\pi^3 \eta L} \sum_{n,odd}^{\infty} \frac{1}{n^3}\left[1 - \frac{\cosh\left(n\pi\frac{y}{h}\right)}{\cosh\left(n\pi\frac{w}{2h}\right)}\right] \sin\left(n\pi\frac{z}{h}\right)$$

In the regime the manuscript is concerned about $(h/w \to 0)$, the above develops an in-plane current density which deviates from the usual parabolic profile to a 'snubbier' current profile.

We agree with the referee that hydrodynamic flow and the corresponding Poiseuille profile crosses over into a 'snubbier' profile according to the formula quoted above if (and only if) the hydrodynamic-to-diffusive crossover is calculated using a reflectivity parameter which encodes either no-slip or partial-slip boundary conditions. Such boundary conditions have represented the go-to for almost all calculations regarding nearly hydrodynamic flows in the last decade.

We would indeed recover this phenomenology in our approach whenever $Q = 0$ and with small finite ℓ_{ee}. Namely, if $Q = 0$, the Boltzmann equation reverts to the standard form discussed in the literature. Specifically, the emergence of the snubbier profile corresponds to the crossover from hydrodynamic to diffusive flow when the rectangular channel becomes so narrow that the channel width $w \gg h$ is much larger than the effective in-plane mean free path ℓ_{mr} (as calculated for example from the in-plane current density).

However, in the manuscript, we do not consider this limit. Instead, we concentrate on the Gurzhi regime where $w < \ell_{mr}$. That latter case corresponds most closely to what the referee dubbed 'specular out-of-plane BCs' and it does not represent a hydrodynamic-to-diffusive crossover but a hydrodynamic-to-ballistic crossover, where the exact nature of the in-plane boundaries are much less important. As the referee correctly points out in his calculations, both the ballistic and the hydrodynamic profiles which the referee calculated for almost specular out-of-plane BCs are essentially parabolic, and merely feature a different curvature, the precise value of which would be determined by the (in-plane) slip length.

The main result of the present manuscript reveals a new type of hydrodynamic-to-ballistic crossover: We show that the ballistic peak in the distribution function which would normally lead to a ballistic flow, is so much suppressed if angular diffusion from surface scattering is present that the flow is prevented from becoming ballistic. In other words, our main finding is that the phenomenology which was previously suggested to govern the hydrodynamic-to-ballistic crossover is incomplete, because it misses an important source of viscous correlations which can be induced by weak surface scattering. We do not expect that this new crossover will be detectable in the in-plane current profile: As the referee has pointed out themselves, the in-plane current profile is not very sensitive to the distinction between ballistic and hydrodynamic flow.

The new transport regime that we find is therefore not in disagreement with anything that the referee has pointed out, but importantly our results cannot be captured by a formalism which uses only a reflectivity coefficient. Neither can the Poiseuille velocity profile which the referee quoted for the hydrodynamic-to-diffusive crossover describe this regime. Thus, the expressions documented in the literature for the in-plane current densities are not applicable in this new transport regime. In the updated manuscript we have made this point much sharper, and we thank the referee for asking this question, because him asking has helped us to better emphasize the novelty of our results.

Note: of-course the above assumes no-slip boundary conditions, and in-general a kinetic theory vs a pde-hydrodynamic solution would be desirable in the case in question (as the authors rightly point out.) My confusion stems from the following: "How can the authors conclude about the nature of experimental results measuring in-plane current densities, when their analysis rules them out by assumption?"

As mentioned, the in-plane current profile for the channel flow cannot distinguish well between ballistic and viscous flows. Therefore, we refrained from using it as an indicator of hydrodynamic correlations. Instead, we directly construct the effective mean free paths from the distribution function, which is a simple and unambiguous measure for the flow regime.

While a numerical solution to the boundary scattering problem would be desirable, as the referee pointed out himself, this is outside of the scope of the present work, neither is it instrumental for the new crossover regime which we find. To repeat, our main result is that angular diffusion leads to a novel transport regime with a new scaling dependence, which has not been documented before.

We remark in passing that the experimental work on which this theory investigation is based on (Nature 607, 74–80 (2022)), similarly did not invoke the shape of the current profile to detect hydrodynamic flow, neither for the flow in the channel nor in the chamber. Instead, we used the direction of the transverse current component, which reverses sign at a specific opening angle of the chamber, as a (binary) and thus robust indicator of hydrodynamic flow. Indeed, one of the main goals of the experiment was to establish a transport measurement/device geometry which allows to diagnose the flow regime independent from the precise current density profile, which can determine the Gurzhi length quantitatively. This is possible based on whether or not a vortical flow develops in the

chamber for different device geometries.

2. In a similar vein, the authors use an isotropic Fermi velocity in their analysis, claiming that in contrast to the highly-anisotropic Fermi surfaces the Fermi velocity is fairly isotropic in a footnote. This is fine, although calculations showing the distribution of the Fermi velocity in the SI would be more convincing. However, since the Fermi velocity enters the BTE as:

$$\boldsymbol{v}_F \cdot \nabla_{\boldsymbol{r}} f + e\boldsymbol{E} \cdot \nabla_{\boldsymbol{k}} f = I[f]$$

then in-order for their assumption pointed out in 1. above to be justified, they would require instead that $v_y \to 0$, not that it's isotropic in 3D. This is clearly violated in WTe_2 looking at Figure 2.

Here we disagree with the referee, for two reasons: Firstly, the gradients in y-direction are much smaller than in z-direction, which is well justified in the limit $h/w \to 0$. Namely, our analysis holds in the limit that the viscous dissipation due to the bulk e-e interaction is negligible compared to the dissipation coming from the surface scattering mechanism. This is a good approximation because we consider the case that the momentum conserving bulk mean free path is much larger than all other length scales, which implies that the in-plane components of the gradient terms which enter in the Boltzmann equation are negligible when taking the limit $h/w \to 0$.

Secondly, the assumption of a mostly uniform Fermi velocity is not primarily tied to the shape of the Fermi surface, but to how uniform the magnitude of the Fermi velocity is. The exact shape of the Fermi surface matters parametrically, but not for the scaling analysis in terms of the fineness ratio α. Thus, while some numerical estimates might slightly change upon retaining the in-plane gradients, the separation of scales $h/w \to 0$ and the derivations done thanks to it will hold up in this more complicated case as well. We reiterate that we do not assume anywhere that $v_y \to 0$, and in fact our distribution function is a full three-dimensional one, as stated in the main text below Eq. (1).

3. The bulk collision integral used in (1), given by the single-relaxation time approximation $I_0 = |\boldsymbol{v}|(f - f_0)/\ell$ conserves no collisional invariants and thus cannot be used to construct hydrodynamic theories. This is nicely discussed in eq. (9) and Appendix A of de Jong and Molenkamp (Phys. Rev. B 51, 13389).

As it is explained by de Jong and Molenkamp (Phys. Rev. B 51, 13389) in Appendix A, for them (and generally in the absence of magnetic fields, cf. Ref. [48] in the updated manuscript) the relaxation time approximation does conserve the average particle density for a channel flow and one can rewrite the corresponding Boltzmann equation in terms of the difference $f - f_0$.

We agree that I_0 does not conserve momentum. However, as our results demonstrate, short-range correlated surface roughness can serve as a source of momentum-conserving scattering processes with an effective mean free path ℓ_{mc} much shorter than ℓ. Since ℓ is so much larger than all other length scales in the problem, it then makes sense to discuss

momentum conservation on the much shorter length scale of ℓ_{mc}.

We note that the length scales after which a given quantity decays are always calculated from the actual distribution function which solves the Boltzmann equation, therefore it is always possible to define decay rates (or length scales) for all 'collisional invariants' (i.e. up to which distance the respective quantity is conserved). However, generically all invariants are conserved only up to the same distance ℓ*, and no further. It is the speciality of hydrodynamic flows that the collisional invariants starting from the second angular harmonic of the distribution function and higher orders, are much shorter lived than the first angular harmonic, which corresponds to the statement that momentum is much longer lived than the average lifetime of a state. In finite-sized geometries, especially with the complex scattering cross section which we are using, it is therefore not possible to rule out the existence of collisional invariants only by inspection of the bulk scattering integral.*

While this is usually rendered a moot-point due to the symmetry of the channel geometry, the authors need to be more careful in general geometries such as the experimental double-chamber geometry they're referencing. More generally, it would be nice if the authors could include a momentum-conserving term in their bulk collision integral, e.g. using the dual-relaxation time approximation. I understand this will of-course make the analytical solution much harder, so it might be out-of scope.

As we pointed out in the beginning, the main novel finding of our work is that short-range correlated surface roughness can introduce a scale separation between the effective momentum conserving and momentum relaxing mean free paths, even when the bulk momentum conserving scattering is irrelevant. This statement does not rely on a specific in-plane geometry. As mentioned, we therefore chose a channel flow geometry with almost specular in-plane boundaries, which makes the in-plane gradients negligible and allows us to separately discuss the out-of plane component of the distribution function.

We agree with the referee that for a future project, it would be interesting to perform numerical calculations for more complicated geometries, but it is indeed out of scope for the present manuscript.

Minor points

I believe the term 'para-hydrodynamics' is unnecessarily confusing, and would urge the authors to remove it. As illustrated above with the velocity profile of an ideal fluid in a rectangular channel, the geometry let alone the boundary conditions naturally affect the observed current densities. To the extent that "electron hydrodynamics" is used to describe electron flow that's "fluid-like" the term "hydrodynamics" should be taken to quantify the extent to which momentum is conserved in the system.

The para-hydrodynamic regime is distinguished by its dissimilar scaling behavior, and the resulting flow is outside of the hydrodynamic-to-ballistic crossover reported so far anywhere in the literature. Since we obtain a macroscopically viscous flows out of a microscopically ballistic calculation, we feel that it is appropriate to refer to this flow

as para-hydrodynamic (i.e. viscous flow which is unrelated to bulk interactions). Additionally, by referring to this flow as para-hydrodynamic, we make the connection to our previous experimental work (Nature 607, 74–80 (2022)), where the same term was used both in the abstract and in the discussion. In the latter case, no criticism against the name "para-hydrodynamics" was raised, and we wish to link this theory manuscript specifically to the aforementioned experimental work.

Please include ϕ in your schematic in Figure 1a.

We have added ϕ in Fig. 1.

Please use a different notation than for your modified Gurzhi parameter in (10). Currently the two sentences immediately after (10) are rather confusing.

Per the referee's request, we changed the symbol in Eq. (10) to D'.

The discussion in the last results section Effective mean free paths is a bit too high-level. Please explain how the "nature" of the transport regime can be reconstructed by inspecting the distribution function more clearly (I found myself digging through references [48,54] to grasp this), especially since the distribution function is geometry dependent?

We have previously shown (Ref [48] in the updated manuscript) that if the two-dimensional distribution function describing the flow in a narrow channel is decomposed into it's angular harmonic components, keeping only the first and second angular harmonics, the Boltzmann equation is simplified to the Stokes-Ohm equation for hydrodynamic flow. If higher angular harmonics beyond second order are present in the distribution function, this is signaling the presence of additional long-lived modes in the flow, something which is known to happen for a ballistic distribution function. In the manuscript, we make use of these observations for the calculation of the momentum relaxing and momentum conserving mean free paths: The estimate for the momentum relaxing mean free path is based on the first harmonic of the distribution function, i.e. we determine it in a way which is agnostic about the presence of absence of higher angular harmonics in the distribution function. Then, we estimate the influence of higher harmonics, corresponding to ℓ_{mc}^{-1} by evaluating the maximal collision rate based on the geometry of the channel. This estimate quantifies which value ℓ_{mc} would assume for a distribution function which is approaching a cosine form (and thus free of high angular harmonics). Both these estimates are asymptotically correct because the high angular harmonics of the distribution function are subleading as a function of α and thus do not contribute in the limit $\alpha \to 0$.

A much less technical way of making these statements is given in the manuscript: We basically decompose the distribution function into two fluids, a hydrodynamic fluid (which features quick relaxation rates in the high angular harmonics and thus has a cosine-shaped distribution function; and a ballistic fluid which is entirely dominated by

high angular harmonics (i.e. it contains the spiky parts of the distribution function).

In the updated manuscript, we have added a remark which explains the reasoning for the two-fluid approximation.

I believe the divergences in the angular diffusion operator should be discussed in more detail. In particular, approaches to regularize them in numerical implementations (see below) would be useful.

While this is certainly outside the scope of the present work, we agree with the referee that figuring out how to set up a three-dimensional numerical code constitutes an important stepping stone for future works. Let us mention which steps we think need to be taken in order to make such a numerical evaluation: From our experience with the angular diffusion term so far, the main difference between the path taken in the present manuscript and a three-dimensional numerical approach is the saddle point approximation. As we emphasize in the appendix, in our approach we employ a simplified angular diffusion operator which emerges when performing the saddle point approximation under the assumption that the in-plane angle (ϕ) *dependence is regular and can be factored out from the distribution function (i.e. there is no further dependence on* ϕ *left in* $h(z, \theta)$*. On the other hand, if one wanted to also keep irregular dependencies on* ϕ*, the more complicated form of the angular diffusion operator which we quote in the appendix in Eq. S18 is the one which has to be used.*

Proof-of-concept Calculations Guiding Concerns

We appreciate that the referee undertook the effort to calculate the current profiles for various transport regime.

At the same time, as we explained above, current profiles are generically a problematic indicator of the flow regime (cf. Ref. [14]). A weakly parabolic profile could either point towards a hydrodynamic flow with fairly specular boundaries, or towards ballistic flow with very diffusive boundaries. As such, current profiles are not the preferred measure to assess the flow regime. Instead, the knowledge of the distribution function is the much better and more versatile indicator of the flow regime.

Independent of this principal issue regarding current profiles, the measured data from the experiment in Ref. [14] indicates a scenario where the in-plane boundaries constitute almost no-stress boundary conditions with a reflectivity parameter of around 70%. In the perfect no-stress scenario, the in-plane current profile would be completely homogeneous along the width of the channel, irrespective of the scattering properties of the top and bottom surfaces, which would only serve to reduce the total current density, but uniformly. In the partial-slip case, the experimental current profile is therefore only weakly parabolic, with only minor quantitative differences between ballistic and hydrodynamic correlations. For this reason, the determination of the current profile is not helpful in connecting the theory result of the present manuscript with the previous experimental findings, neither in a channel geometry, nor using the chamber geometry.

Thirdly, we emphasize that none of the numerical examples considered by the referee

correspond to the limit considered in our work, because he considers the case where $Q = 0$. As the three depicted cases labeled as "diffusive" "hydrodynamic" and "ballistic" all assume $Q = 0$, they represent the regular hydrodynamic-to-ballistic crossover, which by construction cannot produce the intermediate para-hydrodynamic flow regime. As mentioned, in the limit $Q = 0$, the Boltzmann equation considered in this manuscript becomes purely ballistic, yielding the standard ballistic phenomenology reported before in the literature.

What the current densities calculated by the referee show are these following main effects: The diffusive current profile (blue) is virtually unaffected by the out-of-plane boundaries, because the intrinsic mean free path is just so much smaller than the the device size. In contrast, the hydrodynamic flow (red) depends sensitively on the out-of plane boundary conditions because the momentum-relaxing mean free path is suppressed substantially by the out-of-plane boundary scattering. Most strikingly, the hydrodynamic profile is rendered ohmic once ℓ_{mr} becomes much smaller than w, and resembles the diffusive case (blue). Finally, the ballistic profile changes in similar fashion as the hydrodynamic profile upon adding strong out-of-plane scattering, but to a lesser extent because the effective mean free path (Gurzhi length) in the bulk is much larger. Namely, the bulk Gurzhi length for the red profiles (hydrodynamic) is 0.35, while it is 5 for the green profiles (ballistic).

We reiterate that as long as $Q = 0$, none of the effects discussed in the manuscript are present in the numerical calculation. The most representative case is the dashed green current profile, to which the angular diffusion term stills needs to be added to bring the flow into the para-hydrodynamic regime.

Reviewers' Comments:

Reviewer #2:
Remarks to the Author:
The concerns raised in the first report have been adequately addressed by the authors in their rebuttal. I recommend publication of the revised manuscript in Nature Communications, as I believe the results are very timely and will be of interest to the broad readership of the journal.

Manuscript: “Para-hydrodynamics from weak surface scattering in ultraclean thin flakes” by Y. Wolf, A. Aharon-Steinberg, B. Yan and T. Holder

Response to the referee(s)

Reviewer #2 (Remarks to the Author):

The concerns raised in the first report have been adequately addressed by the authors in their rebuttal. I recommend publication of the revised manuscript in Nature Communications, as I believe the results are very timely and will be of interest to the broad readership of the journal.

Response:
We thank the referee for recommending publication of the manuscript in its current form.
